# Frequency, lethality, and demographic trends of acute respiratory viruses in hospitalized patients: insights from a German tertiary care hospital from july 2022 to april 2023

**DOI:** 10.1186/s12879-025-11521-1

**Published:** 2025-09-16

**Authors:** Juliane Mees, Michael Eisenmann, Anna Höhn, Sina Ebert, Tamara Pscheidl, Nina Roth, Gülmisal Güder, Nils Petri, Isabell Wagenhäuser, Manuel Krone

**Affiliations:** 1 Infection Control and Antimicrobial Stewardship Unit, University Hospital Würzburg, Würzburg, Germany; 2 Department of Anaesthesiology, Intensive Care, Emergency and Pain Medicine, University Hospital Würzburg, Würzburg, Germany; 3 Department of Internal Medicine I, University Hospital Würzburg, Würzburg, Germany; 4 Department of Paediatrics, University Hospital Würzburg, Würzburg, Germany; 5 Central Laboratory Unit, University Hospital Würzburg, Oberdürrbacher Str. 6, Würzburg, 97080 Germany

**Keywords:** Acute respiratory infections, Respiratory viruses, Lethality, SARS-CoV-2, Influenza, RSV, Surveillance, Infection control

## Abstract

**Background:**

Acute respiratory infections (ARI) exhibit varying lethality rates, influenced by individual and population factors. This retrospective study aimed to analyse infection frequency, clinical characteristics, and factors associated with lethality in hospitalized patients with seasonal ARI pathogens.

**Methods:**

Virological and demographic data of hospitalized patients ≥ 18 years who tested positive for at least one ARI viral pathogen (Influenza, Adenovirus, Coronavirus, human Metapneumovirus (hMPV), Parainfluenza, Rhinovirus, Respiratory Syncytial Virus (RSV) and SARS-CoV-2) were collected from 07/2022 to 04/2023 at a German tertiary care hospital. Logistic regression analysis was used to analyse factors influencing lethality. Univariate comparisons examined pathogen-specific differences in length of stay and lethality.

**Results:**

Among 1,657 hospitalized patients with at least one detected ARI pathogen, 89 (5.5%) passed away. Logistic regression analysis indicated a significant association between advanced age and lethality (OR = 1.05 per year, *p* < 0.0001). Patients infected with ARI pathogens other than SARS-CoV-2 or hMPV exhibited a heightened risk of lethality compared to those with Influenza. While statistical significance was reached only for Adenovirus (OR = 5.99, *p* = 0.049), elevated risk of lethality was also observed among hospitalized patients infected with Coronavirus (OR = 2.22), RSV (OR = 2.18), and more than one pathogen (OR = 2.07).

**Conclusions:**

Lethality rates varied among the examined ARI pathogens. Compared to Influenza, Adenovirus, Coronavirus, and RSV showed elevated lethality rates and an increased risk of intrahospital death among ARI-infected patients. RSV emerged as a notable concern for hospitalized adults. Additionally, age also arises as a significant risk factor for lethality associated with ARI during hospitalization.

**Trial registration:**

This study is a retrospective analysis of fully anonymized routinely collected patient data and does not require registration in a clinical trial registry.

## Background

Individuals of all age groups are affected by acute respiratory infections (ARI) ranging from asymptomatic or mild illness up to severe, fatal outcomes [1, 2]. The lethality of ARI is influenced by various individual and population-based factors [3–5]. These include age or pre-existing diseases which can significantly impact the course of disease [6]. Vulnerable populations such as hospitalized patients face particularly severe consequences, as previous studies on Respiratory Syncytial Virus (RSV) or Influenza have already shown [7–9]. ARI are a leading cause of hospitalization in individuals with compromised immune status. In particular the elderly or those with pre-existing conditions like chronic obstructive pulmonary disease (COPD) [10, 11].

During the COVID-19 pandemic, SARS-CoV-2 took the lead as the most common respiratory pathogen that occurred in hospitals [12]. Although there is already a large body of knowledge on ARI caused by Influenza, RSV and SARS-CoV-2 in adults in the hospital setting, there are currently few studies on ARI in adults that investigate the lethality of other respiratory viruses like Rhinovirus, Adenovirus, Human Coronavirus, and hMPV in the setting of hospital [13]. In addition to the risk for individual patients, ARIs can also burden the healthcare system by affecting healthcare workers. They may suffer from an increased risk of conducting an ARI and the resulting potential of intrahospital transmission chains, nosocomial infections and intrahospital ARI outbreak events [14, 15].

This study aims to investigate factors influencing the lethality of adult individuals infected with ARI pathogens in a post-pandemic hospital setting during the endemic transition to be able to assess effective and appropriate preventive measures.

## Methods

### Study setting

Clinical and demographic data of adult hospitalized patients were recorded from 01/07/2022 to 30/04/2023 as part of the surveillance during the COVID-19 pandemic at a tertiary care hospital in Germany [16, 17]. Screening for SARS-CoV-2 was performed by universal real-time reverse transcriptase-polymerase chain reaction (RT-PCR) screening on admission during the study period on all hospitalized patients, reflecting infection control measures implemented during the COVID-19 pandemic [18]. In Contrast, testing for other ARI pathogens (Influenza, Adenovirus, Coronavirus, hMPV, Parainfluenza, Rhinovirus, and RSV) was performed on specific suspicion based on the constellation of symptoms and the patient’s clinical condition as part of routine clinical diagnostics. Oropharyngeal swabs of the patients were sent to the hospital’s virology department to perform RT-PCR. RT-PCR was conducted using eight distinct, validated protocols, as previously described and published and listed in the supplementary materials of Wagenhäuser et al., 2024 [19]. In case of positive RT-PCR diagnosis of an ARI pathogen, the case was added to the hospital’s ARI database for surveillance purposes. This study is a retrospective analysis of these existing clinical and laboratory data; no additional diagnostic procedures were initiated for study purposes.

Inclusion criteria for the patients were:

- Age ≥ 18 years.
- Hospitalization.
- Tested positive during hospitalization for at least one ARI: Adenovirus, hMPV, Parainfluenza, Rhinovirus, RSV, Coronavirus (HCoV-OC43, HCoV-NL63, HCoV-HKU1, HCoV-229E), Influenza (A and B), SARS-CoV-2.

Exclusion criteria:

- Outpatients including patients who were initially admitted as inpatients and discharged on admission day during their stay.
- Missing data on demographic information.

Data were retrieved from the hospital’s electronic medical records system on SAP i.s.h. med^®^ (Walldorf, Germany) and transferred into the hospital’s ARI database based on a Microsoft Access^®^ platform (Redmond, USA).

Based on all infections recorded in the ARI database, the corresponding patient files were manually screened and documented for the following parameters: date of birth, legal gender, date of hospital admission, date of hospital discharge or date of death, and PCR test results on the identified virus(es).

The collected variables included:

1. Age (at the time of hospitalization).
2. legal gender.
3. Length of hospital stay (LOS) in days.
4. Identified type of virus or, if more than one virus has been detected, classification as co-infection.
5. Information on whether the patient has died.

Comprehensive data on pre-existing conditions, vaccination status or comorbidities were not consistently available across cases and were therefore not included in the analysis. Also, treatment-related variables, such as antiviral agents, were not available for standardized assessment.

### Statistics

The data analysis was performed using GraphPad Prism 9.5.1 (GraphPad Software, San Diego, CA, USA). Descriptive statistical methods were employed to summarize the data, including measures of central tendency and dispersion, and to gain insights into the relationships between the variables. Age-specific analyses were performed stratified by the following age groups: 18–30, 31–40, 41–50, 51–60, 61–70, 71–80, 81–90, 91–100 and over 100 years. Univariate comparisons were performed on the pathogen dependency of the LOS using the Kruskal-Wallis-Test and on the lethality using the Chi-squared test. A logistic regression model was employed to evaluate the influence of age, virus type (reference: Influenza), gender (reference: male), and LOS on the likelihood of death. Influenza was selected as the reference category for virus type due to its high frequency and clinical relevance, serving as a standard comparator in respiratory infection research. Male gender was used as the reference category for gender. All variables were entered simultaneously into the model using a forced-entry approach. Categorical variables were dummy coded. The two-tailed significance level α was set to 0.05.

### Use of large language models

Large Language Models (LLMs) were used for language improvement purposes only. The tools ChatGPT (OpenAI, San Francisco CA, USA) and DeepL (DeepL SE, Cologne, Germany) were used.

## Results

**Fig. 1:**
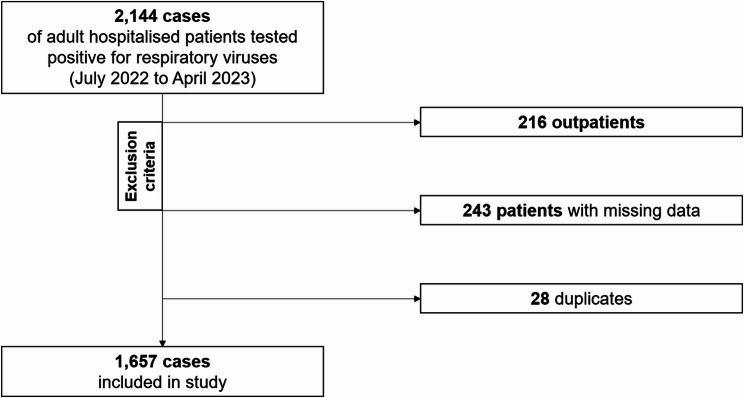
Flowchart for the study inclusion

Within the study period, a total of 2,144 hospitalized adults tested positive for respiratory viruses. Of these, 487 cases were excluded from further analyses following the study protocol leading to a total of 1,657 individuals included (Fig. 1).

### Age and gender distribution

**Fig. 2:**
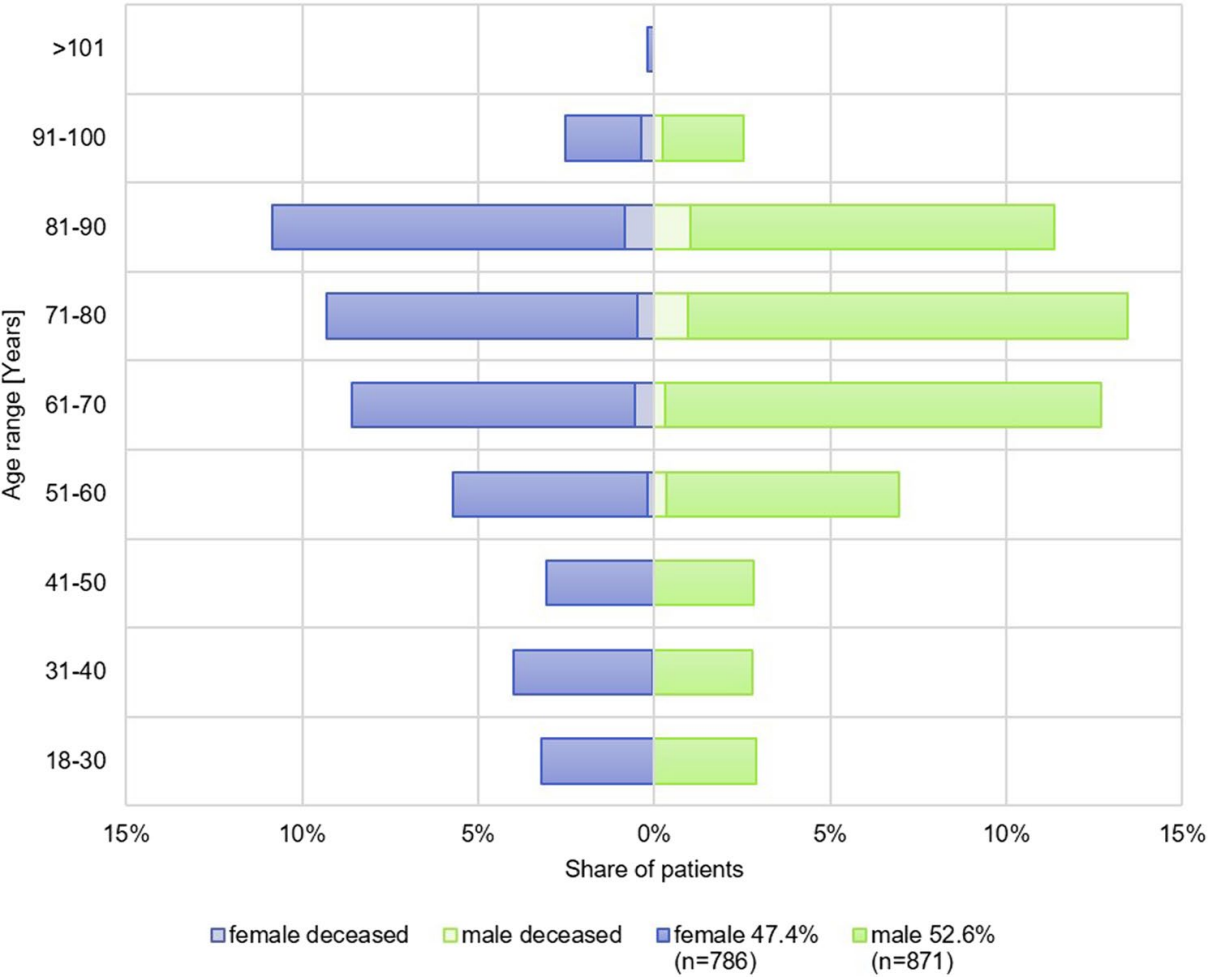
Age distribution of hospitalized patients who tested positive stratified by gender. Percentages are calculated within each age range

Among all included patients 52.6% were male and 47.4% were female (Fig. 2). The median age of patients who tested ARI pathogen positive was 74.0 (IQR: 66.5–85.5) years. The highest ARI prevalence (21.8%, 361/1,657, Fig. 2) occurred in the cohort ages 71–80 years. The median age of deceased adult hospitalized patients with ARI infection was 79.0 (IQR: 69.5–86.5) years.

Male (light green) and female (purple) patients were differentiated. Deceased patients were depicted in light colour.

### Length of hospital stay

**Fig. 3:**
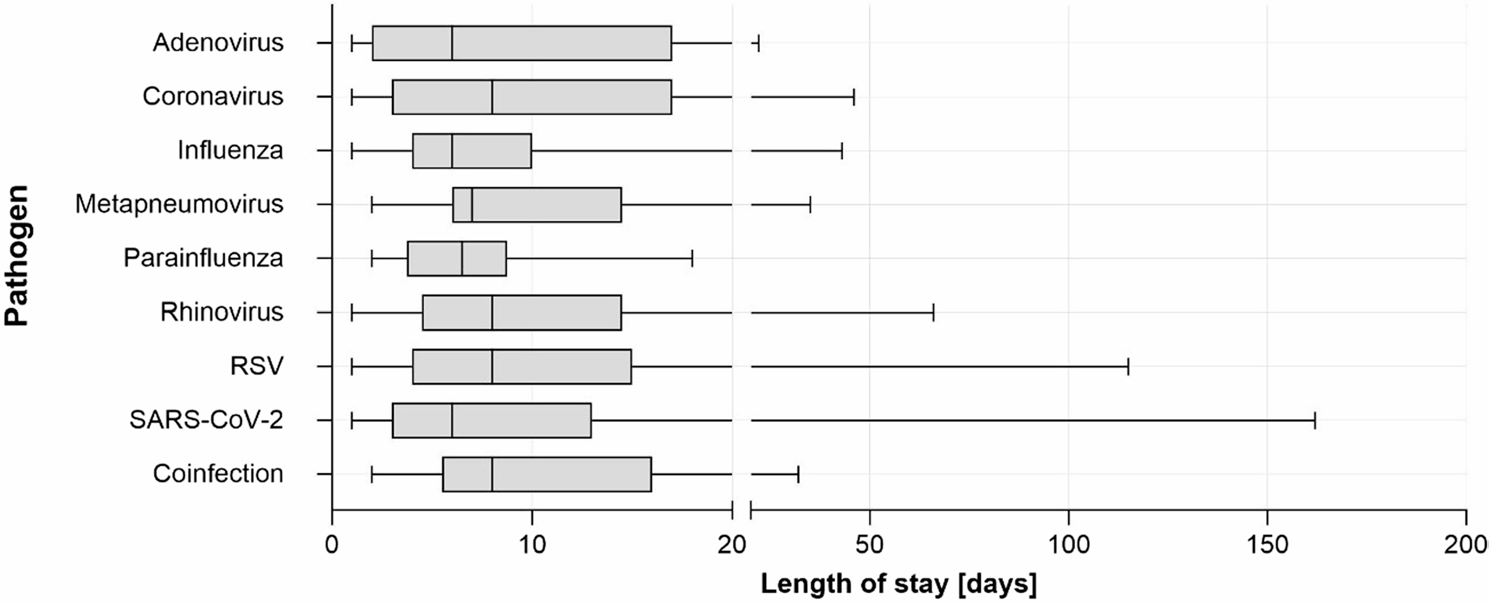
LOS in days of total study population displayed by pathogen

The median LOS of the study population was 7.0 (IQR: 3.0–13.0) days. The total LOS ranged from 1 to 162 days (Table 1; Fig. 3). Stratified by ARI pathogens the median LOS ranged from 6.0 to 8.0 days without statistical significance in the univariate comparison (*p* = 0.19, Kruskal-Wallis-Test). The median LOS of deceased patients was 8.0 (IQR: 3.0-14.5) days, ranging from 1 to 66 days.

**Table 1:** Characteristics of hospitalized patients tested positive for at least one respiratory virus

	overall							deceased							
	Cases (*n*)	Male (*n*)	Female (*n*)	Median age (years)	Age IQR (years)	Median LOS (days)	LOS IQR (days)	Cases (*n*)	Pathogen Lethality (%)	Male (*n*)	Female (*n*)	Median age (years)	Age IQR (years)	Median LOS (days)	LOS IQR (days)
Overall	1,657	871	786	70	(56.0–81.0)	7	(3.0–13.0)	89	5.5	46	41	79	(69.5–86.5)	8	(3.0-14.5)
Adenovirus	13	6	7	50	(37.5–63.0)	6	(2.0–17.0)	2	15.4	2	0	79	(79.0–79.0)	9.5	(6.0–13.0)
Coronavirus	31	18	13	59	(46.0–69.0)	8	(3.0–17.0)	3	9.7	2	1	56	(53.0–79.0)	2	(1.0–17.0)
Influenza	116	50	66	69	(47.0-82.8)	6	(4.0–10.0)	7	6.0	2	5	85	(65.0–90.0)	5	(4.0–9.0)
hMPV	25	13	12	74	(66.5–85.5)	7	(6.0-14.5)	2	8.0	0	2	75.5	(68.0–83.0)	17.5	(6.0–29.0)
Parainfluenza	18	9	9	72	(64.8–77.8)	6.5	(3.8–8.8)	2	11.1	1	1	79.5	(67.0–92.0)	6	(5.0–7.0)
Rhinovirus	57	34	23	65	(52.5–75.0)	8	(52.5–75.0)	6	10.5	4	2	66	(50.5–83.0)	16.5	(3.5–42.0)
RSV	90	40	50	74.5	(62.8–83.3)	8	(4.0–15.0)	13	14.4	8	5	79	(71.0-87.5)	7	(2.0–12.0)
SARS-CoV-2	1,286	691	595	70	(57.0–81.0)	6	(3.0–13.0)	52	4.0	27	25	81	(71.0–87.0)	9	(3.0-14.8)
Coinfection	21	10	11	62	(46.5–75.0)	8	(5.5–16.0)	2	9.5	2	0	62	(51.0–73.0)	11	(10.0–12.0)

*IQR *Interquartile rate, *LOS* Length of stay in days, *hMPV* human Metapneumovirus, *RSV* Respiratory Syncytial Virus

### Types of detected respiratory viruses

The most commonly identified pathogen was SARS-CoV-2 with 1,286 cases (77.6%). The fewest cases were found for Adenovirus with 13 (0.8%) (Table 1).

### Lethality

In total, 89 of 1,657 patients (5.37%) died during their hospitalization. The highest lethality stratified by the virus was observed in patients infected with Adenovirus (15.4%, 2/13). Given the low number of cases, these results should be interpreted with caution. The lowest in patients infected with SARS-CoV-2 (4.0%, 52/1,286) without statistically significant differences in the univariate analysis (*p* = 0.05, Chi-squared test).

**Fig. 4:**
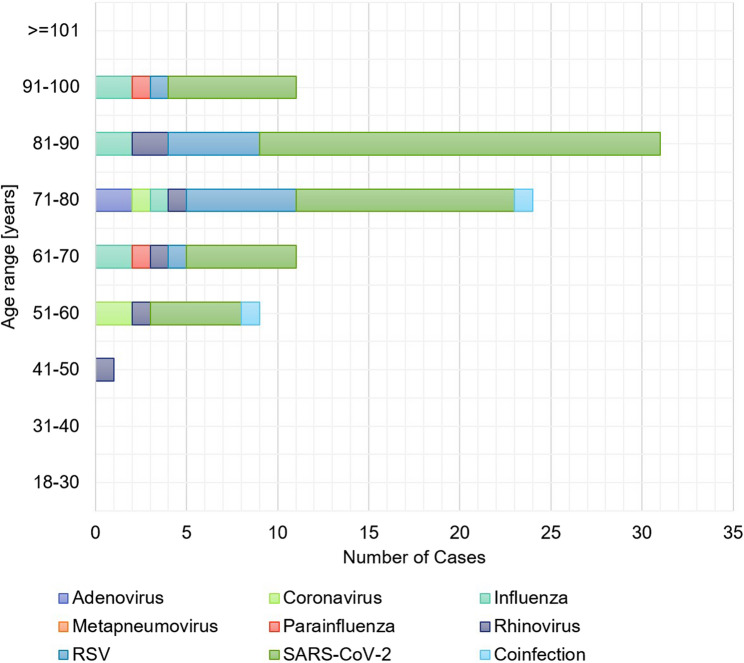
Lethality stratified by age groups (years) and type of viruses

No deaths were recorded in the age groups 18–30 and 31–40 years. The highest number of deaths stratified by virus was identified in the age group of 81 to 90-year-old patients. The proportion of deceased for each age group increased with age: 51–60 years (4.5%, 9/199 cases), 61–70 years (3.5%, 12/341), 71–80 years (7.1%, 35/352), 81–90 years (8.5%, 31/366) and over 91 years (11.1%, 11/99) (Fig. 4).

Lethality of patients infected with Adenovirus (violet), Coronavirus (light green), Influenza (turquoise), hMPV (orange), Parainfluenza (red), Rhinovirus (dark blue), RSV (blue), SARS-CoV-2 (green), and those with Coinfection (light blue) with indication of the proportion of deaths caused by each virus (total of deceased hospitalized patients = 89).

### Logistic regression analysis

**Fig. 5:**
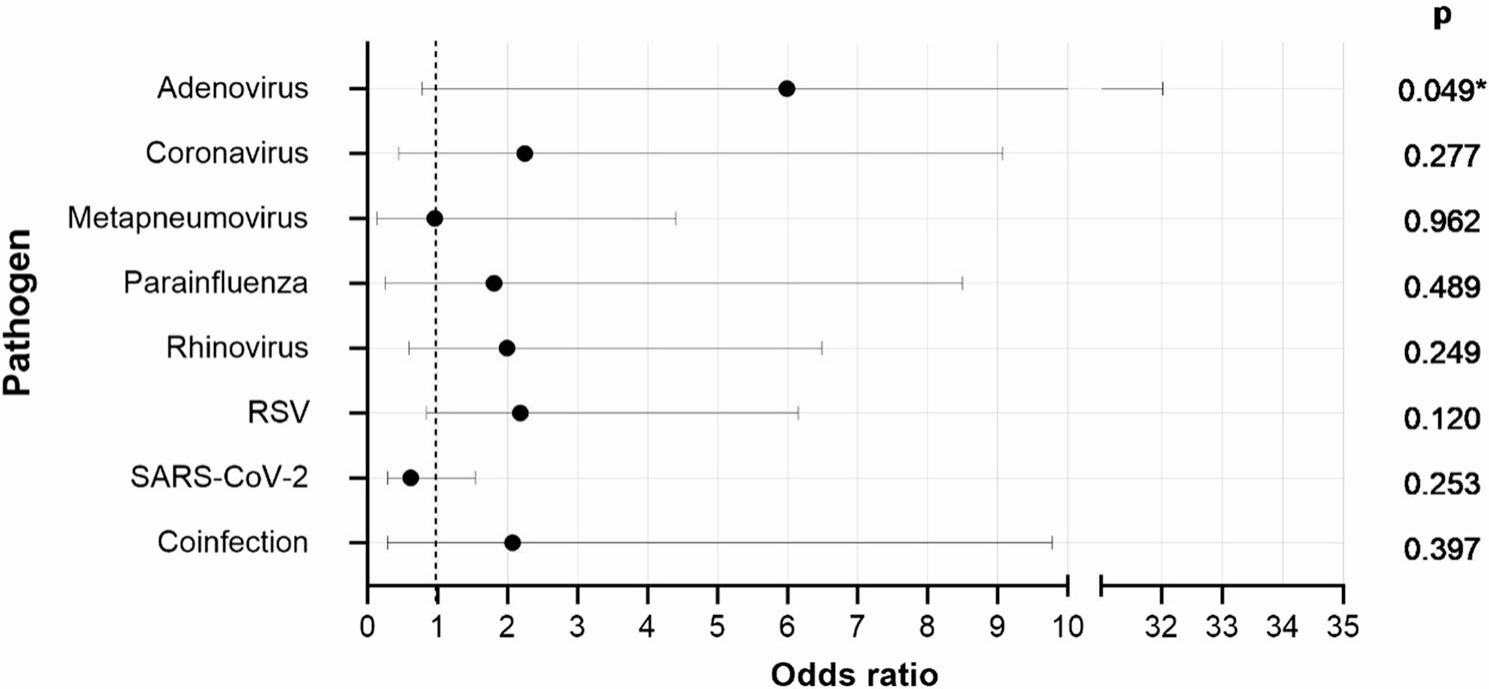
Odds ratios for intrahospital death of different respiratory viruses or coinfection compared to Influenza, with reference line at odds ratio = 1

The results of the logistic regression analysis show that age significantly influenced the lethality rate (OR: 1.046 per year, *p* < 0.001). The analysis showed no significant difference in the lethality rate for the LOS (OR: 1.001, *p* = 0.89) or gender (OR: 0.096, *p* = 0.69). Figure 5 shows the ORs for intrahospital death between different virus types or coinfection (more than one ARI pathogen detectable) compared to Influenza as reference from a logistic regression model including 1,657 adult patients with detection of respiratory virus including the variables age, gender, LOS and detected pathogen.

## Discussion

This retrospective study provides an opportunity to gain insights into the epidemiological situation and lethality rates among ARI-infected hospitalized adults. The most frequently identified respiratory pathogen was SARS-CoV-2, followed by Influenza, RSV, and Rhinovirus. While SARS-CoV-2 was still the predominant ARI pathogen and also caused the most deaths in total, intrahospital lethality was the lowest among the included ARI pathogens. This might be associated with the predominance of the Omicron SARS-CoV-2 VOC and the overall high vaccination rates in the general population, which likely also applies to our patient cohort [20, 21]. In addition, recent research suggests that the clinical severity of SARS-CoV-2 infections has decreased over time due to viral evolution. While earlier variants were linked to higher lethality, later Omicron lineages have been associated with reduced pathogenicity [22]. Alternatively, it may reflect a testing and awareness bias with lower rates of underdetection [20, 21, 23]. The availability and use of antiviral therapies may have additionally contributed to the observed differences in lethality between the respective ARI pathogens. During the study period, specific treatments were available for SARS-CoV-2 and Influenza, while for other ARI pathogens only supportive care was possible [24–27]. These therapeutic options may have mitigated disease severity and improved outcomes for patients diagnosed with SARS-CoV-2 or Influenza.

Among the patients who tested positive for an ARI pathogen during their hospitalization, the highest lethality rate was found in the Adenovirus and RSV cohorts. Due to the low number of cases of Adenovirus, however, these results must be viewed critically. In the logistic regression analysis, compared to Influenza, the highest risk was found for RSV, Adenovirus and Coronavirus.

Age was the only factor that correlated significantly with lethality in ARI-infected hospitalized patients. Infections with ARI pathogens, particularly Influenza or RSV, were diagnosed more frequently in adults over 60 years of age, and lethality also increases for this age group. This finding aligns with previous studies identifying age as a risk factor for ARI-related lethality [7, 23, 28, 29]. The increased susceptibility of older adults to ARI can be attributed to several age-related factors. Immunosenescence, i.e. the gradual decline in immune function associated with advancing age, impairs the body´s ability to develop effective immune responses to viral respiratory pathogens. In addition, older patients often suffer from multiple comorbidities, which exacerbate the clinical course of ARI. Furthermore, physiological changes reduce the ability to eliminate pathogens and recover from respiratory infections [30, 31]. These factors contribute to the increased risk of lethality observed in older patients with ARI.

This study has several limitations: Some ARI pathogens might be underrepresented. Unlike SARS-CoV-2, for which mandatory admission screening was implemented during the pandemic period at the study centre, testing for the other ARI pathogens was only performed based on clinical suspicion, potentially biasing detection towards more severe cases [17]. RT-PCR screening for the remaining respiratory viruses was only performed in case of ARI symptoms and clinical suspicion of an infection other than SARS-CoV-2. Intrahospital and population-based preventative measures in the context of the COVID-19 pandemic, which were also applied during the study period, such as wearing masks may also have resulted in lower numbers of other respiratory viruses compared to SARS-CoV-2 [32]. The presented data capture the lethality of patients infected with an ARI pathogen. No statement can be made about the isolated ARI lethality as to whether their death was caused by the pathogen. The exact cause of death of the patients and comprehensive clinical data on other conditions at the time of hospitalization, including the indication for hospitalization, were not recorded. Furthermore, data on individual COVID-19 vaccination status and pre-existing comorbidities were not available, which limited our ability to perform stratified or adjusted analyses regarding risk factors for severe outcomes. The viruses examined in this study were not subtyped across the board. It was therefore not possible to investigate subtypes of certain types in fatal cases. As in studies with similar study settings it was therefore not possible to distinguish between patients whose death was primarily caused by an ARI or an underlying disease [33]. In addition, data on antiviral treatment were not collected in our study; we were unable to assess their direct impact on lethality. Nevertheless, the unequal availability of targeted treatments should be considered as a relevant confounder when comparing outcomes across ARI pathogens.

## Conclusions

This study highlights the continued clinical relevance of ARIs in hospitalized adult patients, even in the context of the post-pandemic period. Although SARS-CoV-2 remained the most frequently detected ARI pathogen and caused the highest total number of deaths. However, the intrahospital lethality rate among patients with SARS-CoV-2 was lower than among those with other respiratory viruses. Notably, infections with respiratory viruses such as RSV were associated with higher lethality rates, particularly among older hospitalized adults.

Our findings conform a strong association between advanced age and ARI-related lethality reinforces the need for targeted prevention strategies focused on protecting older and vulnerable populations. Moreover, the diagnostic challenge posed by febrile illnesses of unknown origin remains significant, with infectious diseases continuing to be the leading cause worldwide, underscoring the complexity of ARI presentations and the need for comprehensive diagnostic approaches [34]. Given that lethality appears to be driven more by host-related factors than by the specific ARI pathogen, public health efforts should prioritize risk-adapted approaches rather than pathogen-specific interventions alone. These findings support the importance of continuous ARI surveillance and age-stratified risk assessment in informing infection control policies. Further investigation is needed to understand the potential roles of evolving viral variants and vaccination coverage in disease severity and outcomes.

## Data Availability

The datasets analyzed during the current study are available from the corresponding author on reasonable request.
